# Myeloid-specific targeting of Notch ameliorates murine renal fibrosis via reduced infiltration and activation of bone marrow-derived macrophage

**DOI:** 10.1007/s13238-018-0527-6

**Published:** 2018-04-11

**Authors:** Yali Jiang, Yuanyuan Wang, Pengfei Ma, Dongjie An, Junlong Zhao, Shiqian Liang, Yuchen Ye, Yingying Lu, Peng Zhang, Xiaowei Liu, Hua Han, Hongyan Qin

**Affiliations:** 1 State Key Laboratory of Cancer Biology, Department of Medical Genetics and Developmental Biology, Fourth Military Medical University, Xi’an, 710032 China; 2 Department of Nephrology, Xijing Hospital, Fourth Military Medical University, Chang-Le Xi Street #15, Xi’an, 710032 China

**Keywords:** renal fibrosis, Notch signaling, macrophages, heterogeneity, EMT

## Abstract

Macrophages play critical roles in renal fibrosis. However, macrophages exhibit ontogenic and functional heterogeneities, and which population of macrophages contributes to renal fibrosis and the underlying mechanisms remain unclear. In this study, we genetically targeted Notch signaling by disrupting the transcription factor recombination signal binding protein-Jκ (RBP-J), to reveal its role in regulation of macrophages during the unilateral ureteral obstruction (UUO)-induced murine renal fibrosis. Myeloid-specific disruption of RBP-J attenuated renal fibrosis with reduced extracellular matrix deposition and myofibroblast activation, as well as attenuated epithelial-mesenchymal transition, likely owing to the reduced expression of TGF-β. Meanwhile, *RBP-J* deletion significantly hampered macrophage infiltration and activation in fibrotic kidney, although their proliferation appeared unaltered. By using macrophage clearance experiment, we found that kidney resident macrophages made negligible contribution, but bone marrow (BM)-derived macrophages played a major role in renal fibrogenesis. Further mechanistic analyses showed that Notch blockade reduced monocyte emigration from BM by down-regulating *CCR2* expression. Finally, we found that myeloid-specific Notch activation aggravated renal fibrosis, which was mediated by CCR2^+^ macrophages infiltration. In summary, our data have unveiled that myeloid-specific targeting of Notch could ameliorate renal fibrosis by regulating BM-derived macrophages recruitment and activation, providing a novel strategy for intervention of this disease.

**Electronic supplementary material:**

The online version of this article (10.1007/s13238-018-0527-6) contains supplementary material, which is available to authorized users.

## Introduction

Renal fibrosis is a common pathological process in end-stage chronic kidney diseases (CKD) including chronic glomerulonephritis, diabetic nephropathy, and ureteral obstruction (Vernon et al., 2010). The dominant feature of renal fibrosis is excessive deposition of extracellular matrix (ECM) in kidney interstitium, which is deteriorated by inflammation, myofibroblast accumulation, and tubular atrophy (Cochrane et al., 2005). So far, no specific therapy has been established to cure renal fibrosis, due to poorly defined cellular and molecular mechanisms of this disease.

Macrophages play pivotal roles in renal fibrosis (Vernon et al., 2010; Wang and Harris, 2011), as depletion of macrophages ameliorates murine renal fibrosis induced by unilateral ureteral obstruction (UUO) (Kitamoto et al., 2009), suggesting that macrophages could serve as a therapeutic target. However, extensive observations have suggested that some macrophages exhibit pro-fibrotic activities, while others play an anti-fibrotic role (Kluth et al., 2004; Wang et al., 2007; Henderson et al., 2008). This controversial is reminiscent of recent findings unveiling the ontogenic heterogeneity and functional plasticity of macrophages (Wynn and Vannella, 2016). Macrophages in adult tissues are constituted by resident macrophages established during embryogenesis, and monocytes-derived macrophages recruited from bone marrow (BM). Most resident macrophages persist in tissues life-long and replenish themselves through self-renewal. Monocytes, on the other hand, are mobilized after tissue injury depending on chemotaxis signaling, and recruited to the inflammatory sites, where they differentiate into macrophages. Macrophages in tissues are activated and polarized into different subpopulations depending on local immunological milieu (Ginhoux and Guilliams, 2016). In response to IFN-γ and/or LPS, macrophages adopt the M1 activation and produce TNF-α, IL-1β, and NO, leading to enhanced inflammation, Th1-biased immune response and exacerbated tissue injury. In contrast, upon treatment with IL-4/IL-13, macrophages are polarized into M2 activation characterized by the up-regulated expression of IL-10, TGF-β, ARG1, and YM1. M2 macrophages exhibit anti-inflammatory activities and Th2 response, which promote tissue repair and remodeling (Vernon et al., 2010; Wynn and Vannella, 2016). In renal fibrosis, it has been reported that M1 macrophages exert pro-fibrotic role whereas M2 macrophages are anti-fibrotic (Nishida et al., 2005; Wang and Harris, 2011). Nevertheless, M2 macrophages express high level of TGF-β, a pro-fibrotic cytokine which promotes ECM deposition and epithelial-to-mesenchymal transition (EMT), leading to renal fibrogenesis (Miyajima et al., 2000). Unveiling the role of these different populations of macrophages in renal fibrosis is a prerequisite of macrophages-targeted therapy.

A number of signaling pathways regulate macrophage recruitment, activation and proliferation. The CCL2-CCR2 signaling is essential for the emigration of monocytes from BM into blood stream (Kitagawa et al., 2004; Seki et al., 2009). M-CSF/c-fms and GM-CSF/CSF2R pathways support the proliferation of resident macrophages in response to altered microenvironment (Le Meur et al., 2002; Hashimoto et al., 2013). In addition, several cell-intrinsic pathways, such as p38/MAPK-, c-Jun/JNK-, and ERK-mediated signaling, have been implicated in macrophages recruitment and activation (Han et al., 2008; Ma et al., 2009). The Notch pathway constitutes contact-mediated cell-cell signaling (Artavanis-Tsakonas et al., 1999). Notch activation is initiated by γ-secretase-dependent cleavages of Notch receptors, liberating the Notch intracellular domain (NIC) that translocates into nuclei to associate with the recombination signal binding protein-Jκ (RBP-J). This event leads to the transactivation of downstream genes that are responsible for cell proliferation and differentiation (Chen and Al-Awqati, 2005; Hu and Phan, 2016). Notch signaling is critically involved in macrophage differentiation and activation in different disease models (Wang et al., 2010; Zhang et al., 2010; Xu et al., 2012; Franklin et al., 2014; Zhao et al., 2016). Recently, our data have shown that disruption of RBP-J in macrophages ameliorates hepatic fibrosis by attenuating inflammation through cylindromatosis (CYLD) in mice (He et al., 2015). However, owing to the complicated roles of Notch in macrophage differentiation and activation, it is valuable to determine the role of Notch signaling in macrophages during renal fibrogenesis. In this study, we show that myeloid-specific Notch signaling significantly modulate renal fibrogenesis as in liver. However, in contrast to hepatic fibrosis, Notch signal regulates macrophages with distinct mechanisms in renal fibrosis, namely the CCR2-mediated monocyte recruitment and local macrophage activation. Collectively, our study indicated that targeting Notch signal in macrophages may be a new therapeutic strategy for kidney fibrosis.

## Results

### Myeloid-specific RBP-J deficiency attenuated UUO-induced renal fibrosis

We first established UUO-induced renal fibrosis in normal mice. Masson’s staining and hematoxylin and eosin (H&E) staining showed that renal fibrosis was induced 1 week or 2 weeks after UUO (Fig. S1A and S1B). Meanwhile, immunofluorescence staining indicated that the infiltration of macrophages increased significantly in obstructed kidney as compared with the contralateral kidney (Fig. S1C), consistent with previous reports (Kitamoto et al., 2009; Vernon et al., 2010; Wang and Harris, 2011).

Notch signaling is critically involved in macrophage activation (Wang et al., 2010; Zhang et al., 2010; Xu et al., 2012; He et al., 2015; Zhao et al., 2016). To determine the role of Notch signaling in macrophages during renal fibrosis, *RBP-J-floxed* mice were crossed with *Lyz2-Cre* transgenic mice to obtain macrophage-specific *RBP-J* knockout (*Lyz2-Cre*/*RBP-J*^*f*/*f*^, *RBP-J* cKO) and control (*Lyz2-Cre*/*RBP-J*^*+*/*f*^, Ctrl) mice according to our previous study (He et al., 2015). Quantitative (q) PCR using genomic DNA from sorted kidney macrophages showed that the efficiency of *RBP-J* deletion was almost complete in the *RBP-J* cKO mice (Fig. S2A and S2B). Myeloid development was not altered apparently in the *RBP-J* cKO mice (data not shown) (He et al., 2015).

**Figure 1:**
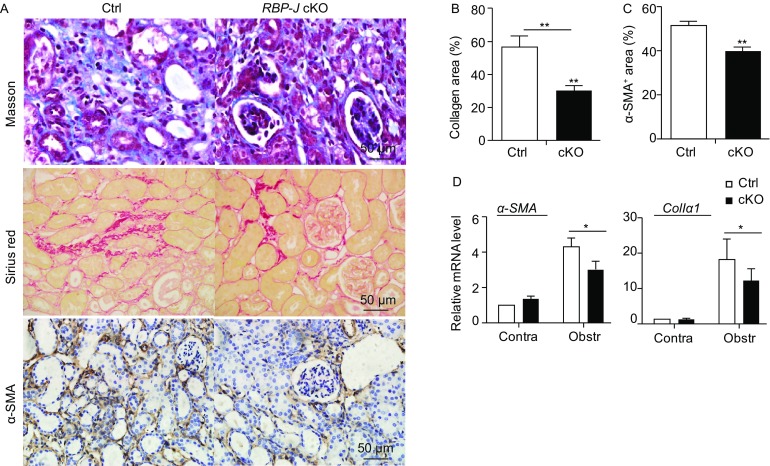
**Myeloid-specific RBP-J deficiency attenuated UUO-induced renal fibrosis**. (A) The *RBP-J* cKO and control mice were subjected to UUO. Masson and Sirius red staining, and anti-α-SMA immunohistochemistry staining of kidney sections were performed 2 weeks after the operation. (B) The collagen-positive areas in (A) were quantified and compared. (C) The α-SMA-positive areas in (A) were quantified in the *RBP-J* cKO and control mice and compared. (D) The kidneys from the *RBP-J* cKO and control mice were analyzed for the relative mRNA expression of α*-SMA* and *ColIα1* 2 weeks after UUO using RT-PCR. Bars = mean ± SD, *n* = 6. *, *P* < 0.05, **, *P* < 0.01

Renal fibrosis was induced by UUO in the *RBP-J* cKO and control mice. Masson’s staining and Sirius Red staining on day 14 after the operation indicated that renal fibrosis was markedly reduced in the *RBP-J* cKO mice: the interstitial collagen area in the fibrotic kidney of the *RBP-J* cKO mice was about half of that in the control (Fig. 1A and 1B). Consistently, the expression of α-smooth muscle actin (α-SMA), a marker of myofibroblasts (Lin et al., 2009), decreased remarkably in the fibrotic kidney of *RBP-J* cKO mice as determined using immunohischemistry staining (Fig. 1A lower panel and 1C). We then examined the mRNA level of α*-SMA* and *collagen I* using qRT-PCR, and found that the expression of both molecules was significantly reduced in the fibrotic kidney of *RBP-J* cKO mice as compared with the control (Fig. 1D). These results indicated that myeloid-specific disruption of *RBP-J* resulted in attenuated renal fibrosis in mice.

### Inhibited expression of pro-fibrogenic factors and reduced EMT in the kidney of *RBP-J* cKO mice after UUO

**Figure 2:**
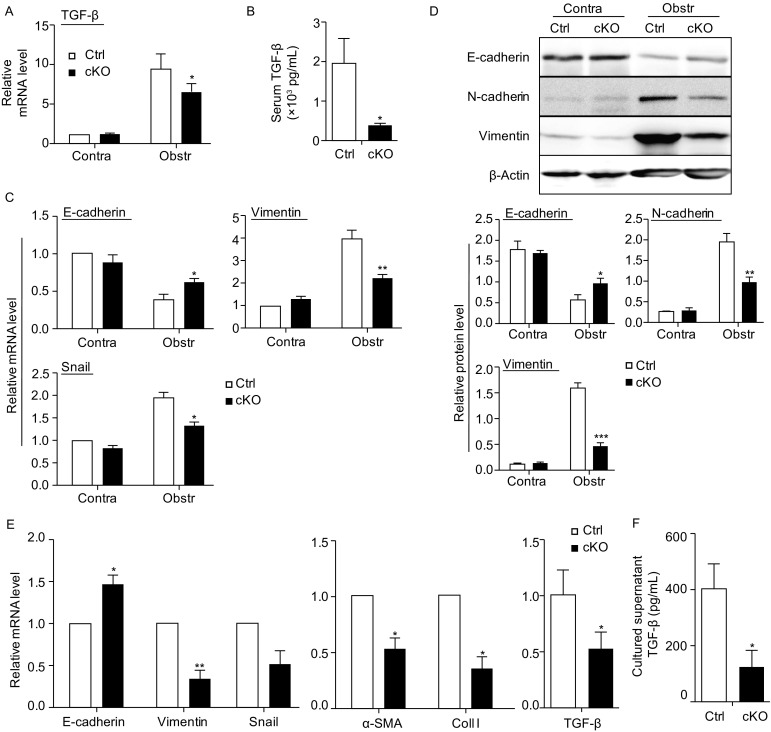
**Decreased expression of TGF-β and EMT in the kidney of myeloid-specific RBP-J deficient mice after UUO**. (A) Relative mRNA expression of *TGF-*β in kidney from the *RBP-J* cKO and control mice was determined 2 weeks after UUO using RT-PCR (*n* = 6). (B) The level of TGF-β in serum of the *RBP-J* cKO and control mice was determined with ELISA (*n* = 6). (C) Relative mRNA expression of EMT-related markers in the kidney was determined by RT-PCR (*n* = 6). (D) The protein level of EMT-related markers in the kidney extracts was detected by Western blotting, and quantitatively compared between groups (*n* = 6). (E) Primary proximal tubular epithelial cells were isolated from normal mice, and cultured for 24 h in the presence of the conditional medium (CM) from kidney macrophages from the *RBP-J* cKO or control mice suffering from UUO for 2 weeks. And then the co-cultured tubular epithelial cells were harvested for detecting mRNA expression of EMT- and fibrosis-related markers using qRT-PCR (*n* = 3). (F) the protein level of TGF-β was detected in conditional medium from cultured kidney macrophages of fibrotic kidney of *RBP-J* cKO and control mice by ELISA (*n* = 3). Bars = mean ± SD. *, *P* < 0.05, **, *P* < 0.01, ***, *P* < 0.001

TGF-β is one of the major cytokines promoting renal fibrogenesis through EMT (Zavadil and Böttinger, 2005; Böttinger 2007; Kalluri and Weinberg, 2009). Therefore, we examined TGF-β expression in the kidney of the *RBP-J* cKO and control mice subjected to UUO using qRT-PCR and ELISA. The results showed that TGF-β was markedly down-regulated in the fibrotic kidney of the *RBP-J* cKO mice as compared with the control (Fig. 2A and 2B). Then, we evaluated EMT in the fibrotic kidneys of the *RBP-J* cKO and control mice by determining the expression of the EMT-related markers using qRT-PCR and Western blotting. The result showed that, compared with the control, the epithelial marker E-cadherin was higher whereas the mesenchymal markers Vimentin and N-cadherin was lower in the fibrotic kidney of the *RBP-J* cKO mice as compared with that of the control (Fig. 2C and 2D). The expression of Snail, a transcription factor driving EMT, was also lower in the fibrotic kidney of the *RBP-J* cKO mice (Fig. 2C). Therefore, myeloid-specific deletion of *RBP-J* ameliorated renal fibrosis through, at least in part, the attenuated TGF-β expression and EMT in kidney.

To further verify whether RBP-J deficiency influenced the ability of macrophages to induce EMT, we cultured primary proximal tubular epithelial cells (PTEpiC) of wild-type mice with the conditional medium (CM) collected from cultured kidney macrophages that were sorted from fibrotic kidney of the *RBP-J* cKO or control mice for 24 h. The phenotype and purity of sorted kidney macrophages was shown in Supplementary Figure 3A. The morphology of the tubule cells cultured with the control CM became fusiform, similar to activated fibroblasts, whereas the tubule cells cultured with the *RBP-J* cKO macrophage-derived CM kept cobblestone-like appearance of normal tubule cells (Fig. S3B). After collecting co-cultured PTEpiC, the mRNA expression of EMT- and fibrosis-related markers was examined using qRT-PCR. The result showed that the mRNA level of *E-cadherin* increased while that of *Vimentin*, *Snail*, α*-SMA*, *collagen I* and *TGF-β* decreased in PTEpiC cultured with the *RBP-J* cKO macrophages-derived CM compared with the control CM (Fig. 2E). Meanwhile, the expression of TGF-β in cultured supernatant of *RBP-J* cKO macrophages was decreased significantly as compared with the control (Fig. 2F). Taken together, these results suggested that RBP-J deficient macrophages exhibited reduced capacity of inducing EMT of tubular epithelial cells.

**Figure 3:**
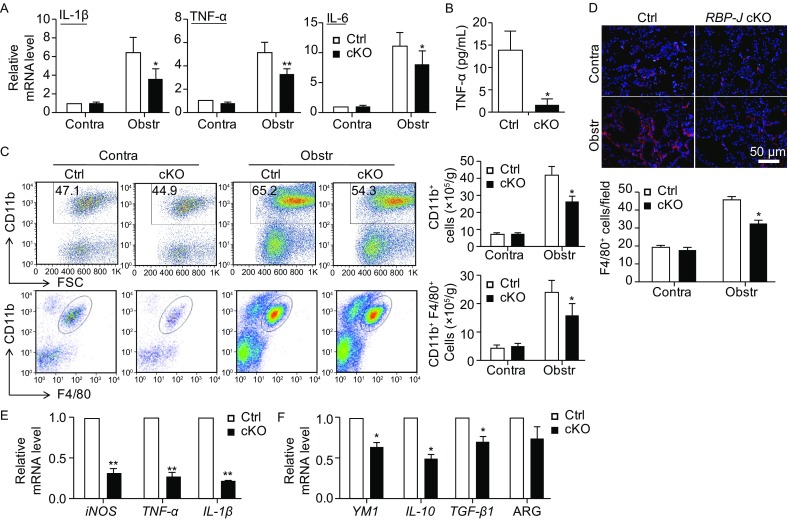
**Ameliorated inflammation and macrophage infiltration and activation in fibrotic kidney of myeloid-specific RBP-J deficient mice after UUO**. (A) *RBP-J* cKO and control mice were subjected to UUO. The mRNA expression of *IL-1β*, *TNF-α* and *IL-6* in the kidney was determined 2 weeks after UUO by qRT-PCR. (B) The protein level of TNF-α was detected by ELISA in serum of *RBP-J* cKO and control mice in (a). (C) Single cell suspensions were prepared from obstructed and contralateral kidney of *RBP-J* cKO and control mice respectively, and then performed FACS analysis. CD11b^+^ cells (myeloid cells) and CD11b^+^F4/80^+^ cells (macrophages) were quantitatively compared. (D) Kidney sections in (a) were stained with anti-F4/80 antibody using immunofluorescence staining, and the F4/80^+^ macrophages were counted and compared. (E and F) Single cell suspensions were prepared from the kidneys 2 weeks after the UUO, and macrophages (CD11b^+^F4/80^+^) were sorted and then cultured *in vitro* for 24 h. The mRNA expression of M1 (E) and M2 (F) macrophage activation markers was determined using qRT-PCR. Bars = mean ± SD, *n* = 6. *, *P* < 0.05, **, *P* < 0.01

### Decreased macrophage infiltration and activation in the kidney of *RBP-J* cKO mice upon UUO

H&E staining showed reduced infiltration of inflammatory cells in the interstitial regions of the fibrotic kidney of the *RBP-J* cKO mice after UUO, suggesting a compromised inflammation (Fig. S4A). Consistently, the level of inflammatory cytokines including TNF-α, IL-1β, and IL-6 decreased significantly in the fibrotic kidney of *RBP-J* cKO mice as compared with the control (Fig. 3A and 3B).

Next we analyzed myeloid populations in the fibrotic kidney of the *RBP-J* cKO and control mice using FACS (Lin et al., 2009). The total number of infiltrated granulocytes (CD11b^+^Ly6G^hi^) in the fibrotic kidney of the *RBP-J* cKO mice was comparable with that of the control (Fig. S4B). Furthermore, we detected the significantly reduced CD11b^+^ myeloid cells and CD11b^+^F4/80^+^ macrophages in the fibrotic kidney of the *RBP-J* cKO mice than in the control (Fig. 3C). Immunofluorescence staining with anti-F4/80 also showed that F4/80^+^ macrophages decreased obviously in the fibrotic kidney of the *RBP-J* cKO mice (Fig. 3D). These data suggested that attenuated inflammatory response in the fibrotic kidney of *RBP-J* cKO mice likely resulted from reduced inflammatory macrophages infiltration.

To further evaluate the role of Notch signaling in regulating macrophages in renal fibrosis, CD11b^+^F4/80^+^ macrophages were sorted from the fibrotic kidney of the *RBP-J* cKO and control mice, and the expression of macrophage activation markers was determined using qRT-PCR. The results showed that the expression of both of the M1 markers *iNOS*, *TNF-α*, *IL-1β*, and the M2 markers *YM1*, *IL-10* and *TGF-β* was reduced obviously in kidney macrophages from the *RBP-J* cKO mice during renal fibrosis (Fig. 3E and 3F), indicating that macrophages activation was attenuated in the fibrotic kidney of macrophage-specific RBP-J deficient mice. Meanwhile, the reduced TGF-β expression in RBP-J deficient macrophages may responsible for decreased EMT in fibrotic kidney of *RBP-J* cKO mice as shown in Fig. 2.

### Macrophage-specific *RBP-J* cKO did not affect macrophage proliferation

**Figure 4:**
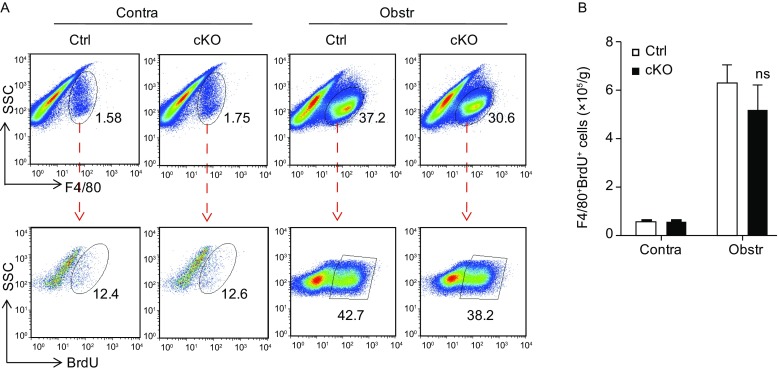
**No significant difference of macrophage proliferation in kidney between** ***RBP-J*** **cKO and control mice after UUO**. (A) *RBP-J* cKO and control mice were subjected to UUO and injected i.p. with BrdU. The BrdU-labeled proliferating macrophages were determined by FACS 7 days after the UUO. (B) F4/80^+^BrdU^+^ cells in (A) were quantitatively compared. Bars = mean ± SD, *n* = 3. ns, not significant

*In situ* proliferation stimulated by local cytokines such as CSF-1 or IL-4 plays an important role in maintaining macrophage homeostasis in tissues (Le Meur et al., 2002; Hashimoto et al., 2013). We then asked whether Notch signaling could influence renal fibrogenesis by regulating macrophage proliferation in kidney. The *RBP-J* cKO and control mice were injected with BrdU and subjected to UUO. FACS analysis showed that while the percentage of proliferating macrophages was significantly higher in the fibrotic kidney as compared with the contralateral kidney, the absolute number of BrdU^+^F4/80^+^ macrophages in the fibrotic kidneys of *RBP-J* cKO mice was comparable with that of the control (Fig. 4A and 4B). This suggested that Notch signaling did not affect local macrophage proliferation in UUO-induced renal fibrosis.

### Notch signaling regulated renal fibrosis mainly through monocytes-derived macrophages

**Figure 5:**
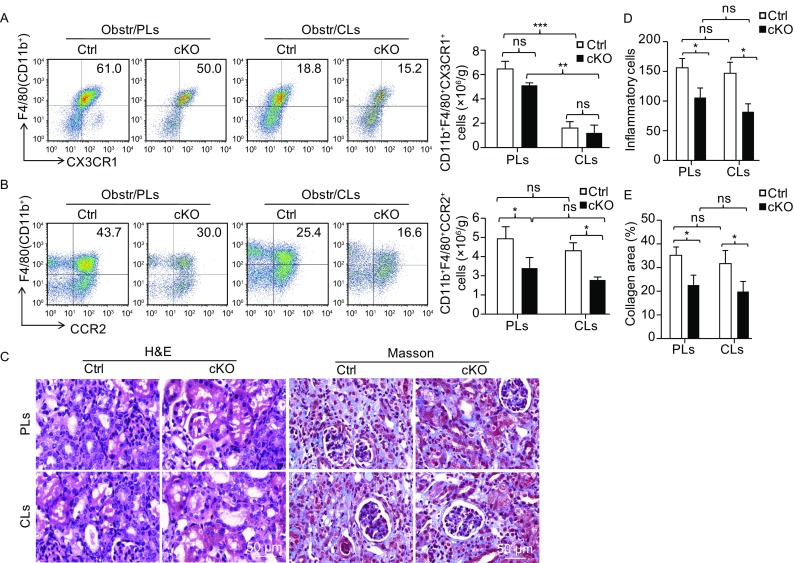
***RBP-J*** **cKO attenuated UUO-induced renal fibrosis independent on kidney resident macrophages**. *RBP-J* cKO and control mice were injected i.v. With liposome-encapsulated clodronate (CLs) or control liposome (PLs) three times before UUO. Kidney macrophages were analyzed by FACS 7 days after UUO, and the number of CD11b^+^F4/80^+^CX3CR1^+^ resident macrophages (A) and CD11b^+^F4/80^+^CCR2^+^ BM-derived macrophages (B) were quantified and compared. (C) H&E staining (left panel) and Masson’s staining (right panel) was performed using kidney sections. (D) The infiltrating inflammatory cells were counted and compared using H&E staining sections in (C). (E) The collagen-positive areas were quantified and compared using Masson’s staining sections in (C). Bars = mean ± SD, *n* = 3. *, *P* < 0.05, ns, not significant

Tissue resident macrophages have been implicated in tissue injury and repair (Yamasaki et al., 2014; Zigmond et al., 2014; Stamatiades et al., 2016). In order to address the role of different origins of kidney macrophages, the *RBP-J* cKO and control mice were injected with liposome-encapsulated clodronate to deplete embryonic-derived kidney resident macrophages before UUO according to published protocol (Kitamoto et al., 2009), and then mice were subjected to UUO for 7 days. FACS analyses showed that the CD11b^+^F4/80^+^CX3CR1^+^ resident macrophages were almost completely depleted by clodronate-liposomes (CLs) treatment (Fig. 5A). In contrast, although the CD11b^+^F4/80^+^CCR2^+^ monocytes-derived macrophages were also affected, this population was still less in *RBP-J* cKO mice than that in control mice irrespective of tissue resident macrophage depletion (Fig. 5B). Meanwhile, upon CLs treatment, the infiltrated inflammatory cells and the degree of renal fibrosis in *RBP-J* cKO mice and control mice did not alter obviously compared with those after liposome treatment (PLs), as shown by H&E staining (Fig. 5C left panel and 5D) and Masson’s staining (Fig. 5C right panel and 5E). These results indicated that Notch signaling regulated renal fibrosis likely through the monocytes-derived macrophages rather than the kidney resident macrophages.

### Myeloid-specific RBP-J deficiency led to reduced recruitment of CCR2^+^ monocytes from BM after UUO

**Figure 6:**
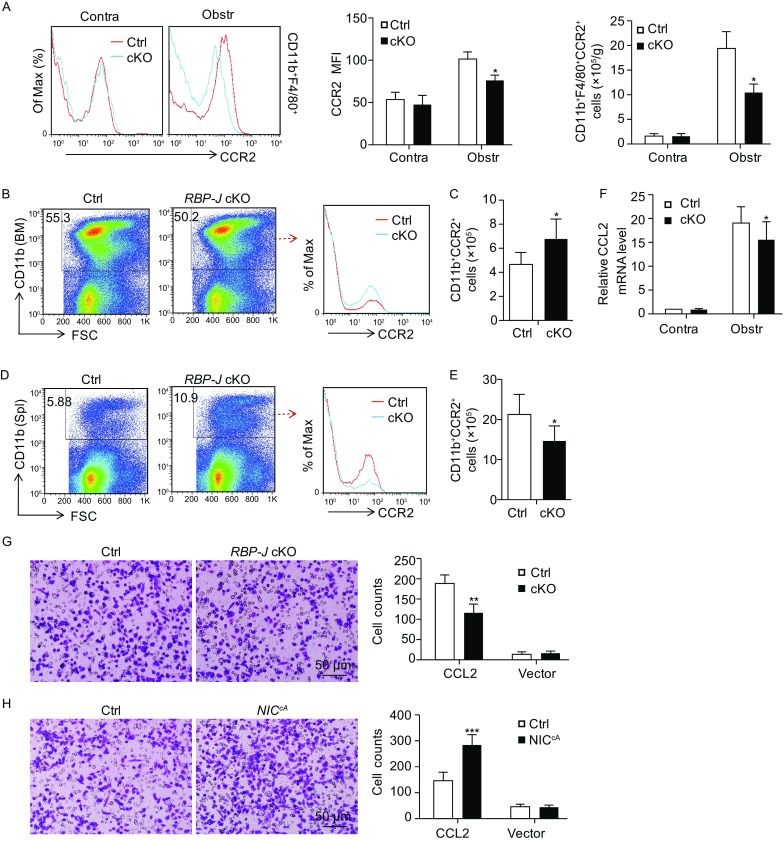
**Myeloid RBP-J deficiency led to the reduced recruitment of CCR2**^**+**^ **monocytes from BM after UUO through CCR2-CCL2 chemotaxis**. (A) *RBP-J* cKO and control mice were subjected to UUO. The expression of *CCR2* in CD11b^+^F4/80^+^ macrophages from obstructed and contralateral kidneys was determined by FACS (right panel). The mean fluorescence intensity (MFI) of *CCR2* expression (middle panel) and the number of CCR2^+^ inflammatory monocytes-derived macrophages were quantitatively compared (left panel) (*n* = 6). (B and C) BM from the *RBP-J* cKO and control mice was collected 2 weeks after the UUO, and the CD11b^+^ monocytes were analyzed by using FACS for the expression of *CCR2* (B). The number of CCR2^+^ inflammatory monocytes was quantitatively compared (C) (*n* = 6). (D and E) Spleenocytes were collected from the *RBP-J* cKO and control mice 2 weeks after the UUO, and the CD11b^+^ monocytes were analyzed by FACS for the expression of *CCR2* (D). The number of CCR2^+^ inflammatory monocytes was quantitatively compared (E) (*n* = 6). (F) The mRNA level of *CCL2* was determined by qRT-PCR in the fibrotic kidney of the *RBP-J* cKO and control mice after UUO (*n* = 6). (G) BMDMs from the *RBP-J* cKO or control mice were plated into the upper chamber of 24-well transwell chambers. The lower chambers were pre-plated for 24 h with HEK293 cells transfected with *CCL2*-expressing or the control plasmid. Cell migration was allowed for 3.5 h at 37 °C, and the migrated cells were quantitatively compared (*n* = 3). (H) BMDMs from the myeloid-specific *NIC* transgenic or control mice were tested for chemotaxis to *CCL2* as in (G) (*n* = 3). Bars = mean ± SD. *, *P* < 0.05, **, *P* < 0.01, ***, *P* < 0.001

Because RBP-J deficiency resulted in reduced inflammatory macrophage infiltration in fibrotic kidney, we next investigated the expression of CCR2, a critical chemokine receptor involved in monocyte migration (Kitagawa et al., 2004; Seki et al., 2009), in kidney macrophages of the *RBP-J* cKO and control mice by FACS. Both the number of CD11b^+^F4/80^+^CCR2^+^ macrophages and the mean fluorescence intensity (MFI) of CCR2^+^ macrophages decreased significantly in the fibrotic kidneys of the *RBP-J* cKO mice (Fig. 6A). FACS analysis also indicated that the number of CD11b^+^CCR2^+^ monocytes increased in the BM but decreased in the spleen of the *RBP-J* cKO mice during renal fibrosis (Fig. 6B–E). Moreover, the mRNA level of *CCL2* in the fibrotic kidney of *RBP-J* cKO mice was also reduced significantly, which might further contribute to the reduced recruitment of CCR2^+^ monocytes into the fibrotic kidney in the *RBP-J* cKO mice (Fig. 6F). These results suggested that the recruitment of inflammatory monocytes from BM, which is mediated by CCL2-CCR2 chemotaxis, was damaged in the *RBP-J* cKO mice undergoing UUO-induced renal fibrosis, likely leading to reduced infiltration of inflammatory macrophages.

To further verify that Notch signaling regulated macrophage infiltration through CCL2-CCR2 chemotaxis, we co-cultured RBP-J deficient or control macrophages with HEK293 cells overexpressing *CCL2* using a transwell system. The result showed that significantly less RBP-J deficient macrophages migrated towards *CCL2*-expressing cells as compared with the control (Fig. 6G). In contrast, when macrophages derived from myeloid-specific *NIC* transgenic mice (Zhao et al., 2016) (*NIC*^*cA*^, see below) were co-cultured with the *CCL2*-expressing HEK293 cells using transwell system, we found that more macrophages with activated Notch signaling migrated towards *CCL2*-expressing cells as compared with the control (Fig. 6H). These data suggested that Notch signaling regulated macrophage infiltration in fibrotic kidney most likely through the CCL2-CCR2 chemotaxis.

Although the MFI of CCR2^+^ macrophages was decreased significantly in the fibrotic kidney of the *RBP-J* cKO mice (Fig. 6A), the mRNA level of *CCR2* decreased slightly in RBP-J cKO macrophages (Fig. S4C), suggesting that Notch signaling might regulate *CCR2* expression on transcription or post-transcription level. Therefore, we isolated monocytes from the BM of the *NIC*^*cA*^ mice, and performed a chromatin immunoprecipitation (ChIP) assay with anti-RNA polymerase (Pol) II or anti-NIC antibody. The result showed that the binding of RNA Pol II to the *CCR2* promoter intended to increase slightly in Notch-activated monocytes, but the occupation of *NIC* on the *CCR2* promoter appeared not changed, suggesting that Notch signaling might activate the transcription of *CCR2* in macrophages indirectly (Fig. S5) or regulate the expression of *CCR2* in post-transcription level that need to be further explored.

### Myeloid-specific Notch activation aggravated UUO-induced renal fibrosis

**Figure 7:**
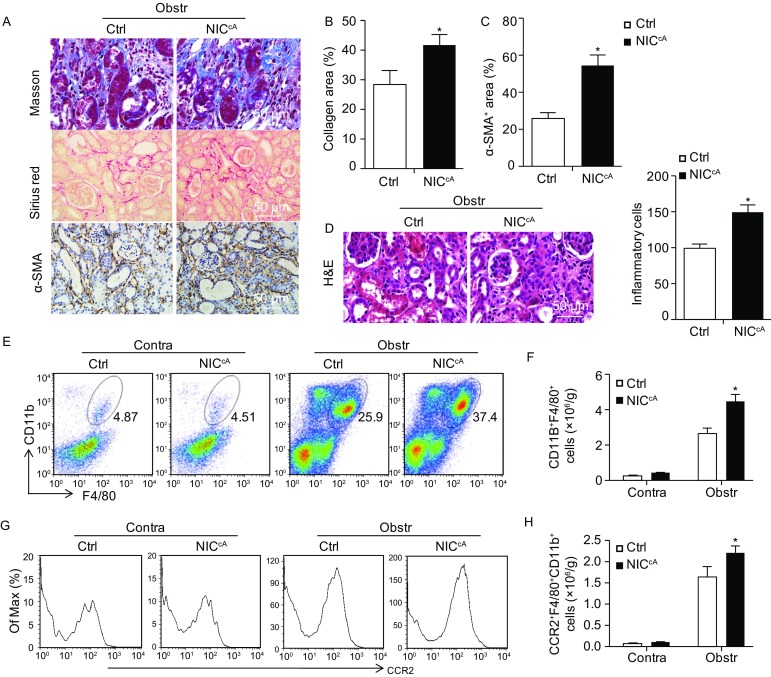
**Myeloid–specific Notch activation aggravated renal fibrosis after UUO**. Myeloid-specific *NIC* transgenic (*NIC*^*cA*^) and control mice were subjected to UUO for 2 weeks. (A–C) Masson (upper panels) and Sirius red staining (middle panel) and α-SMA staining (lower panels) in fibrotic kidney sections was performed (A). The collagen-positive areas (B) and α-SMA^+^ areas (C) were counted and compared. (D) H&E staining of kidney sections were performed 2 weeks after the UUO, and the inflammatory cells in the kidney interstitial (C) were quantified. (E and F) Single cell suspensions were prepared form the fibrotic kidneys. CD11b^+^F4/80^+^ macrophages were determined by FACS (E). The number of CD11b^+^F4/80^+^ macrophages in (E) was quantitatively compared (F). (G and H) CCR2^+^ inflammatory macrophages in (E) were determined by FACS (G), and the number of CD11b^+^F4/80^+^CCR2^+^ macrophages was quantitatively compared (H). Bars = mean ± SD, *n* = 6. *, *P* < 0.05, ns, not significant

So far, our data indicated that disruption of Notch signaling in macrophages ameliorated UUO-induced renal fibrosis by reducing macrophage infiltration and activation. We then set out to confirm the effect of Notch signaling in macrophages on renal fibrosis by using the myeloid-specific *NIC* transgenic mice (Zhao et al., 2016) (Fig. S6). The *Lyz2-Cre*/*STOP*^*flox*^*-NIC* (*NIC*^*cA*^) and control mice (*Lyz2-Cre*) mice were subjected to UUO. Masson’s staining and Sirius Red staining and H&E staining showed that activation of Notch signaling in macrophages aggravated renal fibrosis following collagen deposition, as well as accompanied by increased inflammatory cells infiltration in the kidney interstitium (Fig. 7A, 7B and 7D). Consistently, more α-SMA^+^ myofibroblasts occurred in Notch activated macrophages mice during renal fibrogenesis (Fig. 7A lower panel and 7C). Meanwhile, FACS analysis showed that the infiltration of the CD11b^+^F4/80^+^ macrophages in the fibrotic kidneys of *NIC*^*cA*^ mice increased as compared with the control (Fig. 7E and 7F), and the CD11b^+^ myeloid cells also increased significantly (Fig. S7A). The number of CD11b^+^F4/80^+^CCR2^+^ macrophages, CCR2 MFI in macrophages and the level of *CCL2* all increased in the fibrotic kidney of *NIC*^*cA*^ mice as compared with the control (Figs. 7G, 7H, S7B and S7C). These results suggested that, contrary to the myeloid-specific *RBP-J* cKO mice, myeloid-specific Notch activation aggravated UUO-induced renal fibrosis through promoting CCR2^+^ macrophages recruitment.

## Discussion

Renal fibrosis is characterized by excessive deposition of ECM, which is produced primarily by myofibroblasts. Despite multiple origins of myofibroblasts as revealed by recent studies (LeBleu et al., 2013; Falke et al., 2015), the critical roles of macrophages in the formation and activation of myofibroblasts in renal fibrogenesis have been consensually appreciated (Vernon et al., 2010; Nikolic-Paterson et al., 2014). Recently, BM-derived macrophages are found to undergo the transition into α-SMA-positive collagen-producing cells, namely activated myofibroblasts, during tissue fibrosis, (Wang et al., 2016; Meng et al., 2016). In our study, the expression of α-SMA or collagen I decreased remarkably in the fibrotic kidney of *RBP-J* cKO mice as determined using immunohistochemistry staining or qRT-PCR (Fig. 1). Given that Notch signaling directly regulates α*-SMA* and *collagen I* transcription (Tang et al., 2008; Hu et al., 2014), we could not formally exclude that RBP-J deficiency in macrophages might directly result in decreased production of α-SMA and collagen I derived from macrophages in fibrotic kidney. This possibility is currently under investigation in our laboratory.

Macrophages are activated and modulated by cell debris and molecules bearing the damage-associated molecular patterns (DAMPs) released by injured cells, and by cytokines present in the specific immuno-microenvironment during CKD (Williams et al., 2010). Differentially activated macrophages exert different even contradictory influences on renal fibrogenesis through secreting a wide spectrum of cytokines, growth factors, chemokines, and other inflammatory factors (Ginhoux and Guilliams, 2016; Wynn and Vannella, 2016). Notch signaling is critically involved in macrophage activation (Monsalve et al., 2009; Wang et al., 2010; Zhang et al., 2010; Xu et al., 2012; Zhao et al., 2016). We have recently demonstrated that disruption of Notch signaling by myeloid-specific *RBP-J* knockout attenuated liver fibrosis by compromising macrophage activation through the CYLD-NF-κB pathway (He et al., 2015). In this study, we extended these findings to UUO-induced renal fibrosis, and found that blockade of Notch signaling by myeloid-specific *RBP-J* knockout remarkably ameliorated renal fibrosis. In contrast, renal fibrosis was aggravated by using the myeloid-specific Notch activation mouse model. Therefore, we conclude that Notch activation is likely necessary for macrophage activation in tissue fibrogenesis. To inhibit Notch activation in macrophages may be a new strategy for fibrosis therapy, at least for liver and kidney fibrosis.

Tissue macrophages contain sub-populations with different ontogenies (Ginhoux and Guilliams, 2016; Wynn and Vannella, 2016). The homeostasis of macrophage repertoire in adults is maintained primarily by *in situ* tissue-resident macrophage proliferation and monocyte influx from BM, as well as programmed cell death involving apoptosis and/or necroptosis (Hashimoto et al., 2013; Yamasaki et al., 2014; Zigmond et al., 2014; Stamatiades et al., 2016). We thus questioned the cellular mechanism(s) by which Notch signaling regulated the macrophage repertoire during renal fibrosis. Our BrdU incorporation experiment indicated that local macrophage proliferation might not contributed to the Notch signaling-mediated regulation of macrophages in renal fibrogenesis. Moreover, the depletion of CD11b^+^F4/80^+^CX3CR1^+^ resident renal macrophages exerted no obvious influence on UUO-induced renal fibrosis in either control or myeloid-specific *RBP-J* knockout mice, suggesting that myeloid-specific Notch signaling might not regulate renal fibrosis through kidney resident macrophages. These results reminded us that Notch signaling might regulate monocyte-derived macrophages recruitment for renal fibrogenesis. Indeed, myeloid-specific *RBP-J* knockout decreased while *NIC* over-expression increased the number of CCR2^+^ macrophages in fibrotic kidney, consistent with attenuated or aggravated renal fibrosis in these mice, respectively. This is also consistent with the finding by Lin et al., who showed that depletion of Ly6C^lo^ resident renal macrophages did not affect fibrosis whereas depletion of circulating monocytes and recruited Ly6C^hi^ macrophages ameliorated renal fibrosis (Böttinger 2007; Lin et al., 2009). However, a recent study by Bettie et al has suggested that circulating monocytes-derived macrophages and liver resident macrophages share many common features and might have similar functions regardless of their origin (Beattie et al., 2016). Further investigations employing more precise lineage tracing and gene targeting mice, such as *CCR2*^−/−^ (Yona et al., 2013) and *CX3CR1*^*GFP*^ transgenic (Seki et al., 2009) on the Notch deficient or activated background, are required to elucidate the cellular mechanism(s) for Notch signaling to regulate macrophages in renal fibrosis.

Inflammatory monocytes released from BM are recruited to inflammation sites and then differentiate into macrophages, followed by polarized activation upon local immuno-microenvironment (Yona et al., 2013). Notch signaling has been well demonstrated to participate in terminal differentiation, activation and polarization of macrophages in various disease models (Wang et al., 2010; Williams et al., 2010; Zhang et al., 2010; Xu et al., 2012; Franklin et al., 2014; Zhao et al., 2016). In the UUO-induced renal fibrosis, although it has been reported that different polarized macrophages, especially M1 and M2 macrophages, regulate the development of renal fibrosis by different mechanism (Vernon et al., 2010), in our study it appeared that both M1 and M2 types of macrophage activation were compromised by Notch blockade in macrophages upon UUO, suggesting that Notch signaling regulated macrophages activation, regardless of M1 or M2 macrophages during renal fibrogenesis. This phenomenon is consistent with our previous findings on Notch signaling regulation of macrophage activation in liver fibrosis (He et al., 2015). Recently, in murine liver fibrosis, Ranmachandran et al reports that one kind of new macrophage subsets are identified based on Ly6c expression, which are distinct from the M1/M2 paradigm, suggesting that more functional classification of macrophages subsets should be used to better represent their biology (Ramachandran et al., 2012). Indeed, Lin et al has found that Ly6^hi^ and Ly6c^lo^ macrophages in kidney possess different function during renal fibrogenesis (Lin et al., 2009). Therefore, it might be possibility that Notch signaling regulates macrophage phenotype outside of M1/M2 classification in kidney and liver fibrosis. Functionally, RBP-J deficient macrophages exhibited reduced capacity of inducing EMT of tubular epithelial cells, due to less pro-fibrotic factor TGF-β secretion in the fibrotic kidney and kidney macrophages. In addition to induce EMT, TGF-β is reported to up-regulate the expression of *CCL2* in macrophages and then promote monocyte recruitment and macrophage accumulation (Border and Noble, 1994; Qi et al., 2006). Therefore, it is reasonable to speculate that the less monocyte recruitment and macrophage infiltration in *RBP-J* cKO fibrotic kidney may caused by the reduced TGF-β secretion through down-regulation of *CCL2* expression in macrophages. Moreover, Franklin has reported that inflammatory monocytes are unable to differentiate into tumor associated macrophages in the absence of *RBP-J* by using *CD11c*^*Cre*^*RBP-J*^*f*/*f*^ PyMT mice (Franklin et al., 2014), this result may also partly explain our findings why less macrophages were accumulated in fibrotic kidney in *Lyz*^*Cre*^*RBP-J*^*f*/*f*^ mice.

In summary, our data have unveiled that Notch signaling regulates macrophage in renal fibrosis at two levels, namely the CCR2-mediated monocyte recruitment and the local macrophage activation (Fig. S8). These findings are of potential significance for establishing new therapeutic strategies for renal fibrosis in CKD. However, it should be cautious considering the spatial- and temporal-specific roles of Notch signaling in renal fibrosis. Notch signaling regulates EMT directly in several types of epithelial cells (Li et al., 2013). Notch signaling is also a critical regulator of pericytes, which have been highlighted as an important source of myofibroblasts during renal fibrosis by recent studies (Duffield, 2014; Tattersall et al., 2016). Even in the macrophage compartment, sub-populations of macrophage with different origins and activation avenues likely exhibit different functions in renal fibrosis of different stages (Kitagawa et al., 2004; Nishida et al., 2005; Wang et al., 2007; Henderson et al., 2008). Specifically Notch-targeted therapies in macrophages might be a useful tool to overcome these obstacles for renal fibrosis treatment.

## Materials and Methods

### Animals

Mice were maintained in the specific pathogen free (SPF) condition on the C57BL/6 background. Mice carrying *Lyz2-Cre* transgene (Clausen et al., 1999) (Stock # 019096, The Jackson Laboratory) were crossed with *RBP-J-floxed* (*RBP-J*^*f*^) (Han et al., 2002) mice or the *ROSA-Stop-floxed-NIC* (*STOP*^*f*^*-NIC*, a gift from HL Li) to obtain *Lyz2-Cre*/*RBP-J*^*+*/*f*^ (Contrl) and *Lyz2-Cre*/*RBP-J*^*f*/*f*^ (*RBP-J* cKO) mice (He et al., 2015), or *Lyz-Cre* (Contrl) and *Lyz2-Cre*/*Stop*^*f*^*-NIC* (*NIC*^*cA*^) mice (Zhao et al., 2016). Mice were genotyped by using PCR with the mouse tail DNA as a template. All primers were listed in Table S1. All animal experiments were approved by the Animal Experiment Administration Committee of the Fourth Military Medical University. All institutional and national guidelines for the care and use of laboratory animals were followed.

### The mouse UUO model

The mouse UUO model was established as described (Vielhauer et al., 2001; Chevalier et al., 2009). Briefly, mice were anesthetized with pentobarbital sodium (40 mg/kg) injected intraperitoneally (i.p.). A flank incision was made and the left ureter was ligated with 4-0 silk suture at two points and cut between the ligatures in order to prevent retrograde urinary tract infection. Both of the obstructed (Obstr) and contralateral (Contra) kidneys were harvested on day 7 or 14 after the ureteral ligation for further analyses. At least 6 pairs of mice were analyzed for each assay.

### Isolation of mouse kidney leukocytes and tubular epithelial cells

Kidney cell suspensions were prepared as previously described (Kitamoto et al., 2009). Kidneys were dissected and dissociated in Hank’s balanced salt solution (HBSS) containing 2.0 mg/mL collagenase IV (Sigma-Aldrich, St. Louis, MO) and 200 μg/mL DNase I (Sigma) for 30 min at 37 °C with intermittent agitation. Single cell suspensions were washed twice in HBSS. Following erythrocyte lysis, cells were washed twice again before further analyses.

For the isolation of kidney tubule epithelial cells, C57BL/6 mice (4-weeks old) were anesthetized and sacrificed. Kidneys were immediately removed and placed in ice-cold HBSS. The renal cortices were dissected visually and sliced into pieces of 1 mm in width, and transferred into 10 mL HBSS containing collagenase IV for each kidney. Tissues were incubated at 37 °C with rotating at 70 rpm for 30 min. After that, Dulbeccoo’s modified Eagle’s medium (DMEM) containing 10% fetal bovine serum (FBS) was added to inactivate the enzymes. The tubule cell suspensions were then passed through a 200-mesh sieve to remove tissue debris, followed by centrifuge at 1,200 rpm for 5 min, and resuspended in 10 mL DMEM/F-12 culture media containing 0.01 mg/mL recombinant human epidermal growth factor (rhEGF) and 10% FBS and antibiotics. Cells were cultured in 6-well plates at 37 °C in 95% air-5% CO_2_.

### Flow cytometry

Cells were re-suspended in FACS buffer (PBS containing 2% FCS and 0.05% NaN_2_) and pre-incubated with anti-rat Fc receptor (CD16/32) for 10 min. And then cells were stained with Alexa Fluor 488 anti-mouse F4/80, APC anti-mouse CD11b, PE anti-mouse CCR2, APC anti-mouse CX3CR1, APC anti-mouse Ly6C, Biotin Ly6G and Avidin PE. The detailed information for each antibody was listed in Table S2. FACS analysis was performed using a FACSCalibur^TM^ flow cytometer (BD Immunocytometry Systems, Franklin Lakes, NJ). Data were analyzed with the Flowjo vX.06 software (Flowjo, LLC, Ashland, OR). Cell sorting was performed using a CytoFLEX flow cytometer (Beckman Coulter Life Sciences, IN). Dead cells were excluded by propidium iodide (PI) staining.

### Depletion of tissue resident macrophages with liposome-encapsulated clodronate

Clodronate (Sigma-Aldrich) was encapsulated in liposomes (CLs) as described (Van Rooijen and Sanders, 1994). Liposomes-encapsulated PBS (PLs) was used as a control. Mice were injected intravenously (i.v.) with 200 μL of CLs or PLs on 2 consecutive days, followed by one more injection 48 h after the second one, and then subjected to UUO according to the protocol of macrophage depletion (Kitamoto et al., 2009).

### *In vivo* labeling with bromodeoxyuridine (BrdU)

Mice were subjected to UUO, and injected i.p. with BrdU (1.2 mg/25 g of body weight) 2 h after the operation. The same BrdU injection was repeated every two days until the mice were sacrificed on day 7. The kidney leukocytes were isolated and stained for BrdU incorporation in macrophages with APC anti-mouse BrdU (Biolegend) and Alexa Fluor 488 anti-mouse F4/80 for further FACS assay.

### Cell culture and transfection

BM-derived macrophages (BMDMs) were isolated and cultured as previously described (Wang et al., 2010). In some case, kidney macrophages were sorted from the fibrotic kidney of *RBP-J* cKO and control mice by FACS, and then the sorted macrophages were counted and equal number of macrophages (1 × 10^5^) from the *RBP-J* cKO and control mice were cultured in 48-well plate for 12 h, followed by collection of supernatants as macrophage-derived conditional medium (CM) for further study.

The coding region of the murine *CCL2* cDNA was amplified by PCR using primers (Forward: GCGAATTCAATGCAGGTCCCTGTCATGCTTCT, Reverse: GCGTCGACCTAGTTCACTGTCACACTGGTCA) with a mouse cDNA library as a template. The *CCL2* gene was inserted into *pCMV1-Flag* to construct *pCMV1-Flag-CCL2*. HEK293 cells (ATCC) were cultured in DMEM (Invitrogen) supplemented with 10% FBS, 2 mmol/L L-glutamine, 100 U/mL penicillin and 100 µg/mL streptomycin. For transfection, cells were seeded in 24-well plates and transfected with *pCMV1-Flag-CCL2* or *pCMV1-Flag* using Lipofectamin 2000^TM^ (Invitrogen, Carlsbad, CA) following the manufacturer’s protocol. Cells were used for further experiments 24 h after the transfection.

### Cell migration assay

BMDMs (1.5 × 10^5^) were plated in the upper chamber of the transwell chambers with an 8 µm polycarbonate filter (Millipore, Darmstadt, Germany) in DMEM with 10% FBS. HEK293 cells (1.5 × 10^6^) transfected with *pCMV1-Flag-CCL2* or *pCMV1-Flag* were plated in the lower chamber. Cell migration was allowed for 3.5 h by incubation at 37 °C, and macrophages migrating to the lower chamber were counted under a microscope. Five randomly selected fields were counted as migrating cell number of each insert if not specified. Every experiment was repeated for at least three times with triplicates.

### Histology

H&E staining and Masson’s trichrome and Sirius Red and α-SMA staining were performed following standard protocols (He et al., 2015). The area of fibrosis after Masson’s staining and Sirius Red staining, and α-SMA^+^ area were analyzed using the WinROOF image processing software (Olympus, Tokyo, Japan). At least 10 digitized images of the renal cortex were analyzed for each sample, and the percentage area of positive staining per field was evaluated. Images were taken under a microscope (BX51, Olympus) with a CCD camera (DP70, Olympus).

For immunofluorescence, tissue sections (2–3 µm cryostat sections) were prepared according to standard procedures. The primary antibodies included anti-mouse F4/80. The secondary antibodies included biotinylated goat anti-rat IgG (H + L) and Alexa 594 anti-rat IgG. DyLight 488 streptavidin was used for further staining with biotinylated antibodies. The detailed antibody information was listed in Table S2. Nuclei were counter-stained with Hoechst 33258 (Sigma). Images were taken under a fluorescence microscope (BX51, Olympus) or a laser scanning confocal microscope (FV1000, Olympus).

### RNA extraction and qRT-PCR

Total RNA was prepared using the TriZol reagent (Invitrogen) according to the manufacturer’s instructions. After reverse transcription, real-time PCR was performed using the SYBR Premix EX TaqTM II kit (Takara, Dalian, China) and the ABI PRISM 7500 real-time PCR system, with β-actin as an internal control. Primers for each gene were listed in Table S1.

### Western blotting analysis

Western blotting was performed routinely, with primary antibodies against E-cadherin, N-cadherin, vimentin and β-actin. Horseradish peroxidase (HRP)-conjugated goat anti-rabbit IgG and goat anti-mouse IgG were used as the secondary antibodies. The detailed information for each antibody was listed in Table 2.

### Enzyme-linked immunosorbent assay (ELISA)

The serum levels of TNF-α and TGF-β were determined with commercial ELISA kits (eBioscience) following the recommended protocols.

### Chromatin immunoprecipiation (ChIP) assay

The ChIP assay was performed using a kit (Merck Millipore, Billerica, MA) according to the manufacturer’s instructions with monocytes from mouse BM. Freshly isolated cells were treated following the standard protocol, and fragmented chromatin preparations were immunoprecipitated with anti-RNA Pol II, anti-NIC or isotype control antibody. Genomic DNA was extracted from the collected immune complexes and analyzed using PCR with the primers targeting *CCR2* promoter fragments (Table S1).

### Statistical analyses

Images were processed using the Image Pro Plus 5.1 software (Media Cybernetics Inc, Bethesda, MA). Data were analyzed with Graph Pad Prism 5 software, version 5.0. Unpaired Student’s *t* test or paired *t*-test was used for the statistical analyses. The level of significance was set at *P* < 0.05.

## Electronic supplementary material

Below is the link to the electronic supplementary material.

Supplementary material 1 (PDF 1052 kb)
