# Supplementary material for: Myeloid-specific targeting of Notch ameliorates murine renal fibrosis via reduced infiltration and activation of bone marrow-derived macrophage
Source: Protein Cell. 2018 Apr 11;10(3):196–210. doi: 10.1007/s13238-018-0527-6 (PMC6338623; doi:10.1007/s13238-018-0527-6)
Supplement: Supplementary file 1 — Supplementary material 1 (PDF 1052 kb) [file 13238_2018_527_MOESM1_ESM.pdf]

Supplemental Materials

Table S1. Sequences of the primers used for PCR.

Name	Purpose	Sequence
iNOS-F*	RT-PCR	5’-GCAGAGATTGAGGCCTTGTG
iNOS-R*	RT-PCR	5’-GGGTTGTTCTGAACTTCCAGTC
Arg1-F	RT-PCR	5’-AGACAGCAGGGAGGTGAAGAG
Arg1-R	RT-PCR	5’-CGAAGCAAGCAAGGTTAAAGC
TGF-β-F	RT-PCR	5’-GACCGCAACAACGCCATCTA
TGF-β-R	RT-PCR	5’-GGCGTATCAGTGGGGGTCAG
α-SAM-F	RT-PCR	5’-GGACTTTGAAAATGAGATGG
α-SAM-R	RT-PCR	5’-TGATGCTGTTATAGGTGGTT
COLIα1-F	RT-PCR	5’-TTGGAGAGAGCATGACCG
COLIα1-R	RT-PCR	5’-TACGCTGTTCTTGCAGTG
Vimentin-F	RT-PCR	5’-CAGAGAGAG GAAGCCGAAAG
Vimentin-R	RT-PCR	5’-ATGCTGTTCCTGAATCTGGG
E-cadherin-F	RT-PCR	5’-TAACAGGAACACAGGAGTCATCA
E-cadherin-R	RT-PCR	5’-GTGGTGGGATTGAAGATCGG
N-cadherin-F	RT-PCR	5’-AAGAGAGACTGGGTCATCC
N-cadherin-R	RT-PCR	5’-TGAGATGGGGTTGATAATG
TNF-α-F	RT-PCR	5’-CCACTTCACAAGTCGGAGGCTTA
TNF-α-R	RT-PCR	5’-GCA AGTGCATCATCGTTGTTCATAC
IL-1β-F	RT-PCR	5’-TCCAGGATGAGGACATGAGCAC
IL-1β-R	RT-PCR	5’-GAACGTCACACACCAGCAGGTTA
CCL2-F	RT-PCR	5’-CAGGTCCCTGTCATGCTTCT
CCL2-R	RT-PCR	5’-GTCAGCACAGACCTCTCTCT
CCR2-F	RT-PCR	5’-ATCCACGGCATACTATCAACATC
CCR2-R	RT-PCR	5’-TCGTAGTCATACGGTGTGGTG
CCR2 pro-F*	CHIP	5’-GGGTGTGATAGGTTCTATT
CCR2 pro-R*	CHIP	5’-GCTGAGGTCTTTACAGGATT
β-actin-F	RT-PCR	5’-CATCCGTAAAGACCTCTATGCCAAC
β-actin-R	RT-PCR	5’-ATGGAGCCACCGATCCACA
Cre-F	Genotype	5’-CCGGTCGATGCAACGAGTGATGAGG
Cre-R	Genotype	5’-GCCTCCAGCTTGCATGATCTCCGG
RBP-J-F	Genotype	5’-GTTCTTAACCTGTTGGTCGGAACC
RBP-J-WT-R	Genotype	5’-GCTTGAGGCTTGATGTTCTGTATTGC
RBP-J-floxed-R	Genotype	5’-ACCGGTGGATGTGGAATGTGT
NIC-F	Genotype	5’-AAAGTCGCTCTGAGTTGTTAT
NIC-WT-R	Genotype	5’-TAAGCCTGCCCAGAAGACTC
NIC-floxed-R	Genotype	5’-GAAAGACCGCGAAGAGTTTG

*F, forward; R, reverse; Pro, promoter

Table S2. Antibodies used in this study.

Name	Supplier	Clone #	Titration
Alexa 488 F4/80	Biolegend	BM8	1:100
APC CD11b	Biolegend	M1/70	1:400
PE CCR2	R&D Systems	475301	1:100
APC CX3CR1	R&D Systems		1:10
APC Ly6C	Biolegend	HK1.4	1:200
Biotin Ly6G	Biolegend	1A8	1:1000
APC BrdU	Biolegend	Bu20a	1:100
rat anti-mouse F4/80	eBioscience	BM8	1:200
rabbit anti-mouse a-SMA	Boster Bio Tec	1A4	1:100
Rabbit polyclonal to NIC	abcam		1:800
PE streptavidin	eBioscience		1:400
DyLight 488 streptavidin	Vector Lab		1:400
Alexa 594 anti-rat IgG	Sigma		1:400
Cy3 anti-rabbit IgG	Boster Bio Tec		1:100
Biotin anti-rat IgG	Vector Lab		1:400
N-Cadherin Rabbit mAb	CST	D4R1H	1:1000
E-Cadherin Rabbit mAb	CST	24E10	1:1000
Vimentin Rabbit mAb	CST	D21H3	1:1000
HRP anti-Rabbit IgG	CST		1:2000
Anti-mouse β-actin	Sigma	AC-74	1:1000
Goat anti-mouse IgG	Boster Bio Tec		1:2000

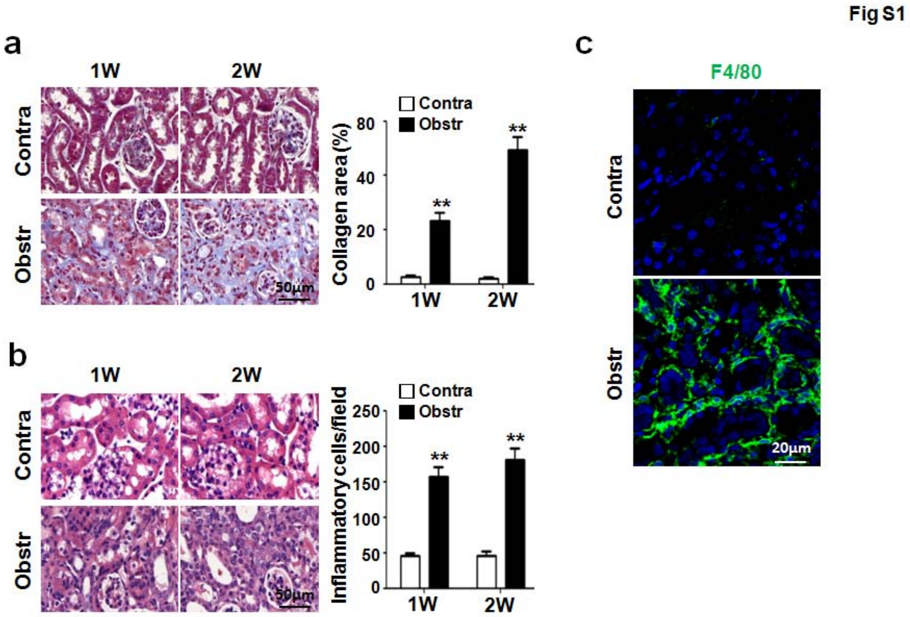

Figure S1. Enhanced F4/80^{+} macrophages infiltration in the fibrotic kidney after UUO. (**a**) C57BL/6 mice were subjected to UUO. The kidneys of the mice were collected 1 or 2 weeks after the UUO, sectioned, and stained by Masson's staining. The collagen-positive fibrotic areas were quantified. (**b**) H&E staining was performed with the kidney sections in (a), and the infiltrated inflammatory cells in the interstitial areas were counted and compared. (**c**) Kidney sections in (a) were stained with immunofluorescence with anti-F4/80 antibodies. Bars = mean ± SD, n = 3. *, $P < 0.05$, **, $P < 0.01$.

Fig S2

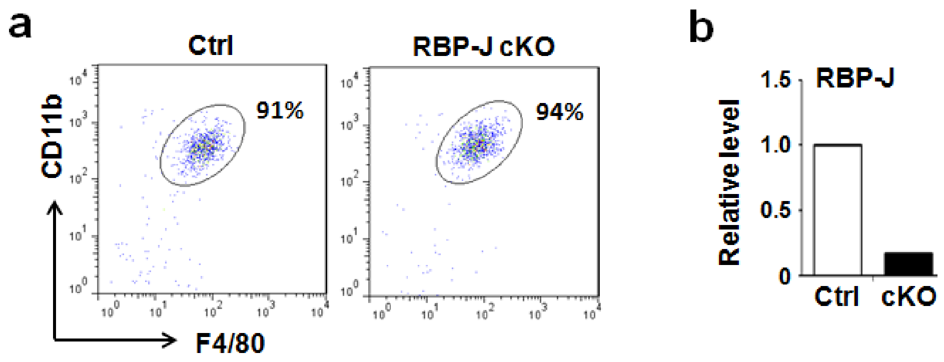

Figure S2. The efficiency of Lyz2-Cre-induced RBP-J knockout in macrophages. (**a**) Kidney macrophages were isolated from the RBP-J cKO and control mice by FACS sorting. The purity of cells was analyzed by FACS after staining with anti-CD11b and anti-F4/80. (**b**) Genomic DNA was extracted from the sorted macrophages. The deletion of the DNA fragments spanning exon 6 to exon 7 of the RBP-J gene was determined using qPCR.

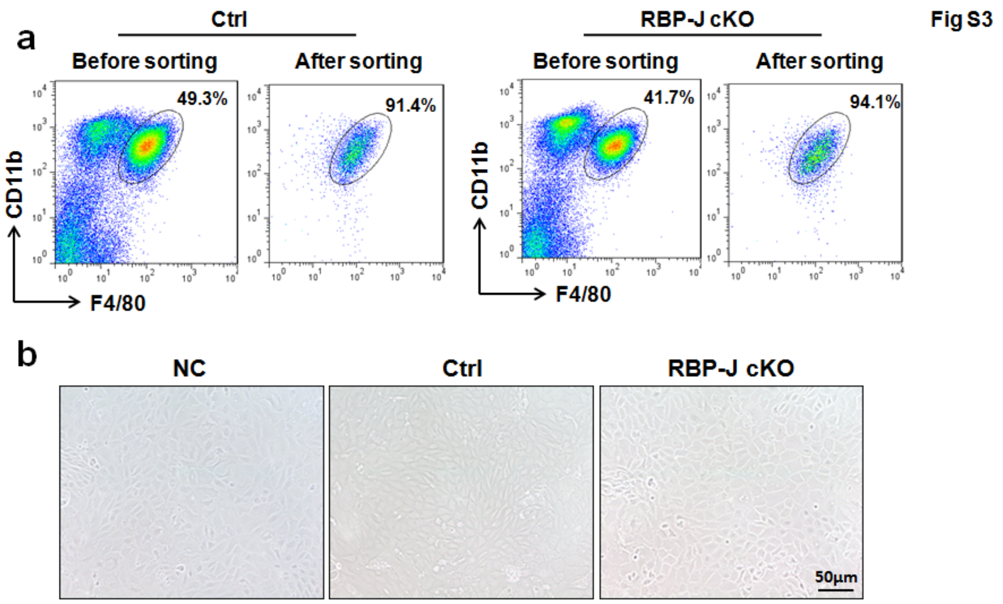

Figure S3. **Sorted kidney macrophages and cultured primary proximal tubular epithelial cells. (a)** Kidney macrophages were isolated from the fibrotic kidney of RBP-J cKO and control mice by FACS sorting. The purity of macrophages was analyzed by FACS after sorting. **(b)** Primary proximal tubular epithelial cells were isolated from normal mice, and cultured for 24 h in the presence of the conditional medium (CM) from kidney macrophages described in a. Pictures were taken under a bright field microscope. Experiments were repeated three times.

Fig S4

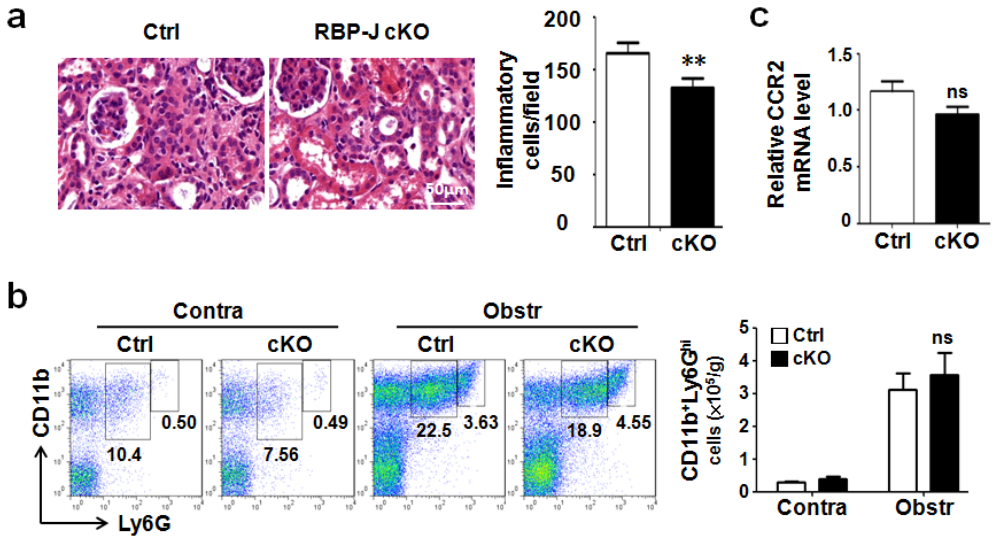

Figure S4. Decreased inflammatory cells infiltration in the kidney of the RBP-J cKO mice after UUO. (**a**) RBP-J cKO and control mice were subjected to UUO. The kidney sections were stained with H&E staining 2 weeks after the UUO. The infiltrating inflammatory cells in the interstitial areas were counted and compared (n = 6). (**b**) Single cell suspensions were prepared from the kidneys in (a), and granulocytes in the kidney were analyzed by FACS with anti-CD11b and anti-Ly6G antibodies. The number of granulocytes in kidney was calculated and compared (n = 6). (c) The mRNA level of CCR2 was determined in sorted kidney macrophages from the RBP-J cKO and control mice after UUO by qRT-PCR (n = 3). Bars = mean ± SD. **, P < 0.01, ns, not significant.

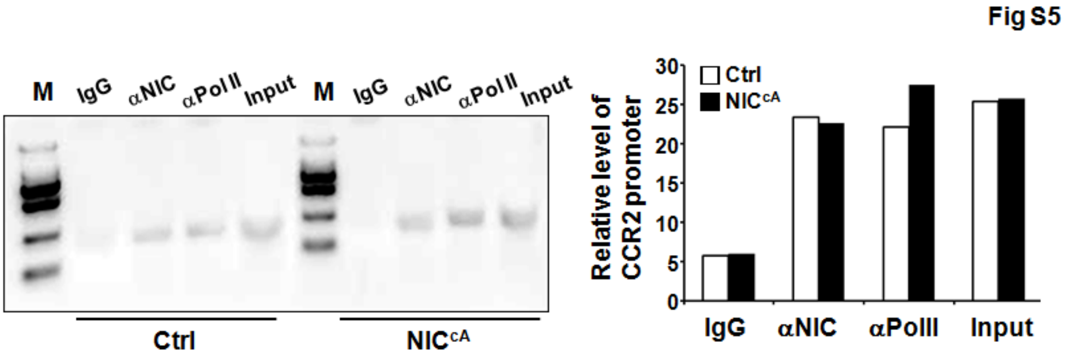

Figure S5. CCR2 was a downstream molecule of Notch signaling in macrophages. ChIP assay. BM monocytes from the myeloid-specific NIC transgenic or control mice were fixed and sonicated, and precipitated with anti-NIC, anti-RNA Pol II, or isotype control IgG. After DNA extraction of the immunoprecipitates, the CCR2 promoter fragments were amplified by using PCR, and analyzed with 3% agarose gel electrophoresis. The bands were quantified with grey-scale scanning and compared. Data represented 3 independent experiments with similar results.

Fig S6

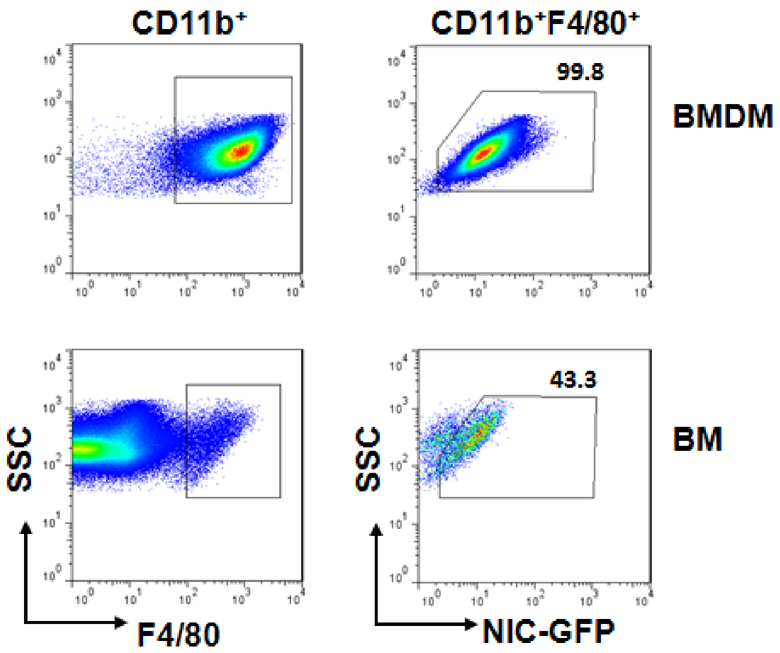

Figure S6. Activation of NIC expression in the NIC^{cA} and control mice. The mice were bred and genotyped as described in the text. The level of NIC in BMDM and total BM cells from the NIC^{cA} mice was evaluated by FACS analysis of GFP, which was co-expressed with NIC through an IRES sequence.

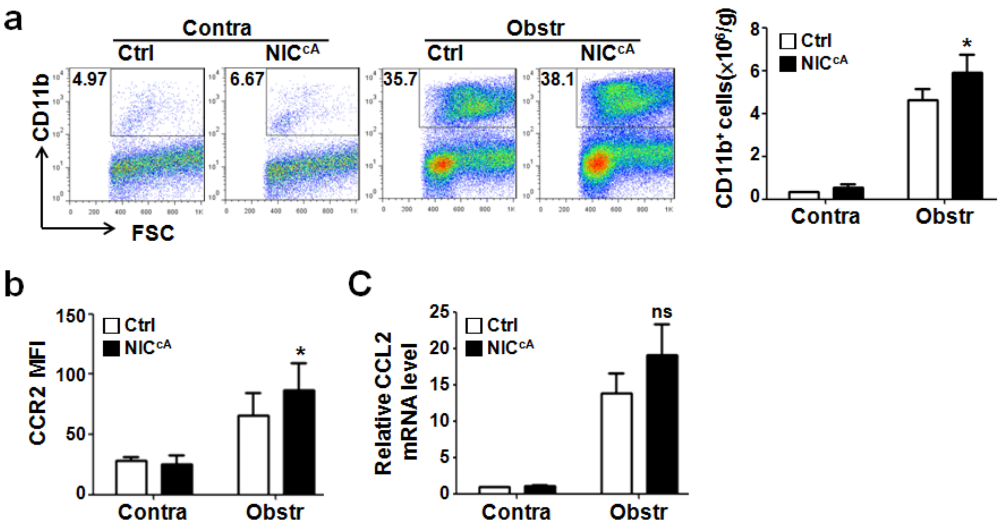

Figure S7. FACS analysis of myeloid cells in the fibrotic kidney of NIC^{cA} mice after UUO. (**a**) The NIC^{cA} and control mice were subjected to UUO. CD11b^{+} myeloid cells in the fibrotic kidney were determined by FACS analysis, and the number of myeloid cells in the kidney was calculated and compared. (**b**) Quantification of the CCR2 MFI in the CD11b^{+}F4/80^{+} cells in the fibrotic kidney of the NIC^{cA} and control mice after UUO (Refer to Figure 7g). (**c**) The mRNA level of CCL2 was determined in the fibrotic kidney of the NIC^{cA} and control mice after UUO by qRT-PCR. Bars = mean ± SD, n = 4. *, $P < 0.05$, ns, not significant.

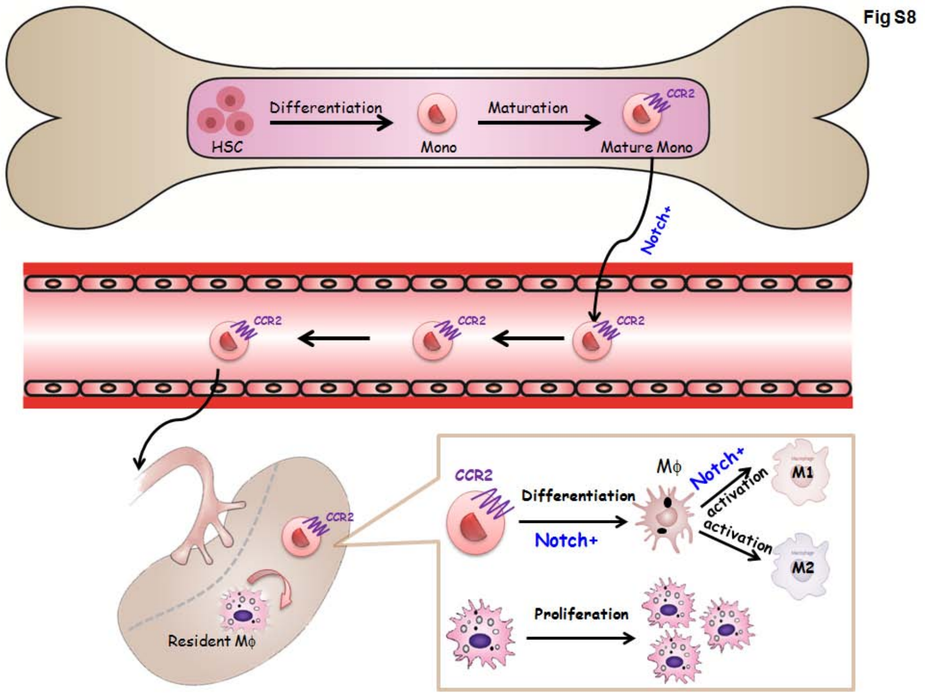

Figure S8. A model of Notch-mediated regulation of macrophages in renal fibrosis. Notch signaling regulates inflammatory macrophages in renal fibrosis at two levels, the CCR2-mediated monocyte recruitment and local macrophage activation. Moreover, according to the published data, Notch may also regulate terminal differentiation of inflammatory macrophages and polarization of activated macrophages, which might participate in certain stages of renal fibrosis. HSC, hematopoietic stem cell; Mono, monocyte; Mϕ, macrophage.
